# Epistasis: Obstacle or Advantage for Mapping Complex Traits?

**DOI:** 10.1371/journal.pone.0012264

**Published:** 2010-08-26

**Authors:** Koen J. F. Verhoeven, George Casella, Lauren M. McIntyre

**Affiliations:** 1 Netherlands Institute of Ecology (NIOO-KNAW), Department of Terrestrial Ecology, Heteren, The Netherlands; 2 Department of Statistics and Genetics Institute, University of Florida, Gainesville, Florida, United States of America; 3 Genetics Institute, Department of Molecular Genetics and Microbiology and Department of Statistics, University of Florida, Gainesville, Florida, United States of America; Johns Hopkins University, United States of America

## Abstract

Identification of genetic loci in complex traits has focused largely on one-dimensional genome scans to search for associations between single markers and the phenotype. There is mounting evidence that locus interactions, or epistasis, are a crucial component of the genetic architecture of biologically relevant traits. However, epistasis is often viewed as a nuisance factor that reduces power for locus detection. Counter to expectations, recent work shows that fitting full models, instead of testing marker main effect and interaction components separately, in exhaustive multi-locus genome scans can have higher power to detect loci when epistasis is present than single-locus scans, and improvement that comes despite a much larger multiple testing alpha-adjustment in such searches. We demonstrate, both theoretically and via simulation, that the expected power to detect loci when fitting full models is often larger when these loci act epistatically than when they act additively. Additionally, we show that the power for *single locus* detection may be improved in cases of epistasis compared to the additive model. Our exploration of a two step model selection procedure shows that identifying the true model is difficult. However, this difficulty is certainly not exacerbated by the presence of epistasis, on the contrary, in some cases the presence of epistasis can aid in model selection. The impact of allele frequencies on both power and model selection is dramatic.

## Introduction

As technology becomes more cost effective, the amount and scale of data available for answering fundamental questions about the underlying genetic contribution to phenotypic outcomes of interest has dramatically increased. Genetic markers, particularly biallelic single nucleotide polymorphisms (SNPs), have been developed for a wide variety of organisms [1], and current SNP discovery techniques and reduced genotyping costs make it feasible to score tens of thousands of markers in many individuals [2], [3], [4], [5].

The plethora of data has sparked interest in developing methodology for hypothesis testing for association mapping. Testing for marker-phenotype associations is done within the context of a specific experimental design [*e.g.* 6], [7], [8]. The experimental design controls the structure of the population under consideration and is therefore a critical component to account for in subsequent modeling and testing. If the population is constructed experimentally, allele frequencies are held constant across loci, and are often equal. When the genetic structure of the population is under experimental control testing marker-phenotype association is often referred to as QTL mapping [7]. In QTL mapping, the issue of which test statistics to use to detect main effects has been discussed quite broadly [*e.g.* 9], [10], [11], [12], [13], [14], [15]. Such one-dimensional genome scans have been enormously popular resulting in more than 5,000 publications. Pedigree-based linkage methods [*e.g.* 16], [17], [18], [19] and some population-based methods [*e.g.* 20], [21] use the knowledge of relationships and transmission of alleles among family members combined with marker-phenotype tests to infer linkage and/or association. As with QTL, the choice of test statistics and approaches is the subject of much research and discussion.

Association mapping is the testing of the null hypothesis that a genetic marker is not associated with a phenotype of interest in an ‘unstructured’ population or rather a population without explicit information on pedigree relations [22], although accounting for relatedness among members of the population due to population substructuring has been shown to be critical [*e.g.* 23], [24]. The development of large numbers of SNP markers has made population based genome-wide association testing increasingly feasible [25], [26], [27], [28].

**Figure 1:**
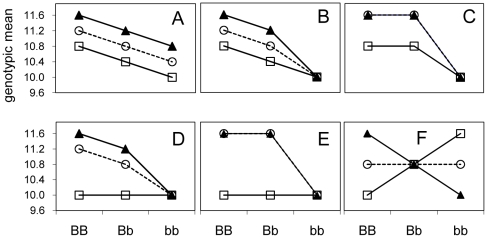
Molecular Epistasis: Two-locus models used in the simulations. The X axis separates the three genotypes at one locus (BB, Bb and bb) while the three lines indicate the different genotypes at the other locus (AA-triangles, Aa-circles and aa-squares). In panel a the effects of both loci are additive (no epistasis). In panel b the effect of the bb combination masks the effect of the A locus - this is an example of recessive epistasis. In panel c recessive epistasis is combined with dominance of the A allele at the A locus, in panel d both the aa and bb combinations exhibit recessive epistasis. In panel e the recessive epistasis of the aa and bb combinations is combined with dominance at the A locus. In panel f, additive-by-additive epistasis, the effects are purely epistatic- under equal allele frequencies this results in the absence of main effects at the A or B locus.

Empirical studies suggest a prominent role for gene interactions (epistasis) in the genetic control of many traits [*e.g.* 29], [30], [31], [32], [33], [34], [35], [36]. The most well understood genetic models for gene interactions are described in terms of qualitative (Mendelian) rather than quantitative traits [37]. In these qualitative trait models gene interactions typically result in masking or covering the effect of some alleles. For example, in an additive model with two biallelic loci there are nine distinct genotypes. In a recessive epistasis model for a qualitative trait, all effects having the combination *aa* for one locus (regardless of the alleles at another locus) have a common outcome. In a quantitative trait model, this corresponds to an equality of means. In this study a series of epistatic cases that are quantitative in nature but based upon such biological or molecular definitions of epistasis are considered. Some of these molecular patterns of epistasis have been observed for quantitative traits [*e.g.* 34]. They are depicted in Figure 1 (b–f). We also consider the additive model (Figure 1a). How these forms of molecular epistasis translate into statistical epistasis, *i.e.* the deviation from additivity in a statistical linear model, depends on allele frequencies at the locus of interest [38], [39]. For instance, in case f (Figure 1f) interactions occur in the absence of main effects when allele frequencies are equal (0.5), but marginal effects arise and can be picked up in one-dimensional genome scans when allele frequencies are different. Thus, the term epistasis has biological interpretations that can be quite distinct from the statistical interaction alone [40], [41]. In addition, typical gene interaction effects that have been described will result in a subset of possible statistical interactions [40].

There is debate about how to model and test for both main effects and interactions when epistasis is present [*e.g.* 32], [39], [42], [43], [44], [45], [46], [47], [48]. In the context of QTL mapping, consideration of molecular interactions among loci has led to constructing models with multiple markers and, using the factor effects construction, testing the main effects and the interaction effects separately [*e.g.* 43], [49], [50], [51], [52]. The power for the detection of the interaction effect alone in the factor effects model can be quite small especially after accounting for multiple testing [53]. In this forward stepwise approach, epistasis is often a nuisance factor that has been assumed to complicate and reduce the efficiency of the QTL mapping exercise [*e.g.* 54].

Recently, it has been suggested that full models be fit during an explicit multiple-locus genome scan [32], [39], [46], [47]. Considering full models (that is, main effects and interactions jointly instead of marginal and interaction effects separately) emphasizes detection of loci rather than partitioning of effects among loci. If epistasis is present, this approach has intuitive appeal from a statistical point of view as the tests of main effects are not readily interpretable in the presence of interaction. Using computer simulations, Marchini *et al.* [47] and Evans *et al.* [39] showed that fitting full models in two-locus genome scans often yields higher detection power than single-locus scans when epistasis is in fact present. This was despite a dramatically lower significance threshold for individual tests after multiple testing alpha adjustment in the pairwise search. In a QTL mapping context for binary traits, Coffman *et al.* [46] evaluated testing full models and showed via simulation that in this context QTL detection power is larger in the presence of epistasis compared to purely additive QTL.

In this paper, the impacts of molecular forms of epistasis on statistical power are explored in an association mapping context. Cases where two loci are jointly responsible for a fixed range of phenotypic variation are studied. The impact of the genetic model (additive or epistatic) on locus detection power is studied. Using a linear models framework, the 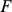 test of the full model and the 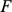 test of the marginal effects are evaluated. The power of the 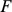 test is shown to be larger under cases of molecular epistasis compared to the additive model. Molecular epistasis also changes the marginal effects and the power for single locus test of association. These expectations are derived under simple assumptions and for equal allele frequencies and fully balanced data. As association mapping data are not likely to be balanced, empirical estimates for the power of association tests under various conditions are derived via simulation. As predicted, the presence of molecular epistasis does, in many cases, have a positive effect on power. When allele frequencies are disparate (that is, when one of the alleles occurs at low frequency) as in many association mapping contexts, *epistasis can dramatically increase power for detection*. Correct model selection is a considerable challenge in the association mapping context, even in cases with relatively high sample sizes. However, the difficulty of the task is not exacerbated by the presence of molecular epistasis.

## Results and Discussion

### Molecular epistasis is expected to increase power

In order to examine the impact of epistasis on power, epistasis needs to be carefully defined. While the term epistasis is common, it has a history of being defined differently by different sets of scientists [40], [41]. In the case of a quantitative character, the definition often used is that of statistical interaction. However, there are many statistical interactions for which no known or plausible biological model may exist [40], [41]. Models of epistasis based upon molecular interactions have been described in terms of qualitative characters [37]. In a qualitative trait, the typical result of epistasis is the masking of one allele's effect by another allele. In a quantitative trait setting this corresponds to equality among means. We translated classic cases of qualitative epistasis into functions of means (Figure 1b–e). Figure 1a represents the completely additive model with no epistasis. Figure 1f represents the case of an interaction when no main effect is present (additive by additive epistasis) and is included here due to it's special status as being often considered as the most diabolical form of statistical interaction.

In addition to this visualization, the genotypic means can be presented in a Punnett square, with the values of the cells representing the genotypic means as in Table 1.

**Table 1:** Theoretical cell means corresponding to the nine genotypes.

	BB	Bb	bb	
AA	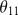	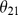	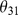	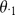
Aa	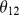	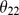	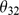	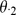
aa	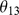	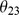	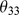	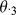
	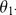	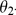	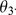	

*Without loss of generality it can be assumed that the values are centered so that the overall mean is zero, that is* 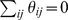 *.*

**Figure 2:**
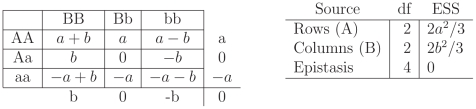
Punnett Square, Factors and Expected Sums of Squares for the additive model (**Fig. 1a**). The left Punnett Square gives genotypic means for the additive model as depicted in Figure 1a. Without loss of generality the genotypic means are centered. The last column and last row are the marginal effects, the average value of the cells in the rows and the columns. The right table gives the Expected Sum of Squares for the 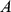 effect, 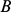 effect, and interaction.

When genotypic values are unconstrained, 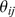 can take on any value. Particular genetic models dictate a relationship among the 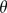 parameters, or in statistical parlance, a constraint in the parameter space. Consider the case of simple additive effects (Figure 1a). The additive genetic model states that the values of the genotypic means (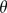 parameters) are constrained as follows: the heterozygote must be the average of the two homozygotes
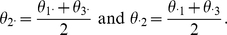
In addition, the two loci are independent. In such a case the values of the 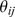 are constrained by this relationship. Without loss of generality, the means can be centered, and the values of the theta parameters represented by 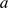 and 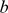 such that the relationship among the 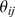 induced by the genetic model are more obvious. The left panel of Figure 2 represents the theta values as they are constrained by the additive model.

For the additive model, the 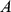 and 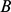 locus are independent. Looking at the statistical table representing the expected sums of squares (ESS) for the additive model (Figure 2) the effect of the Rows(A) and the effect of the Columns (B) do not influence each other. This can be seen by the absence of 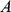 effects in the 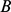 ESS and *vice versa*. The ESS are directly related to the power of the 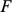-test (see the Methods Section) in that larger values of ESS result in larger power. For example, for the 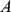 effect:
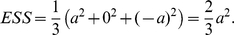
As the effect 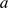 increases, the ESS will be larger and the detection power will be greater. Similarly, there is greater power to detect a column effect 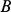 if the value of 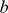 increases (Figure 2). In the additive model, there is no interaction among the row and column effects therefore no epistasis; no matter the value of 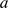 and 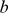 the ESS for epistasis is zero. The total ESS are 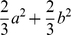.

**Figure 3:**
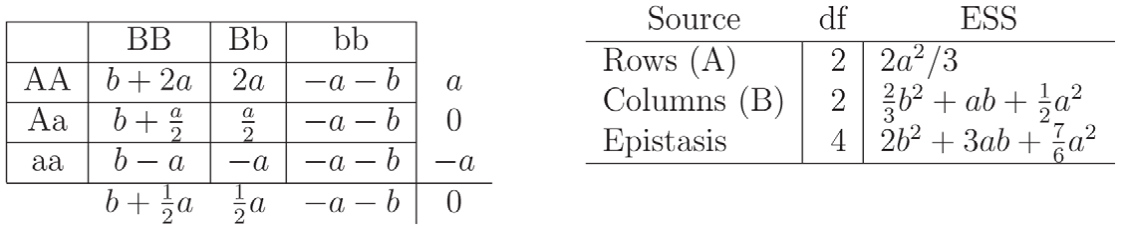
Punnett Square, Factors and Expected Sums of Squares for recessive epistasis (**Fig. 1b**). The left Punnett Square gives genotypic means for the recessive epistasis case as depicted in Figure 1b. The last column and last row are the marginal effects, the average value of the cells in the rows and the columns. The right table gives the Expected Sum of Squares for the 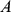 effect, 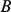 effect, and the interaction.

Molecular forms of epistasis (Figure 1b–f) can be visualized for quantitative traits as the restriction of the parameter space, that is equality among means. For example, in recessive epistasis (Figure 1b), 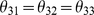. A particular example of this case is given in Figure 3.

In the case of recessive epistasis, the Punnett square in Figure 3 makes it clear that the marginal effect of the 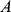 locus will have an impact on the marginal effect of the 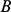 locus. More formally, in the examination of the ESS (Figure 3), the row effect 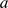 is present in the ESS for the Column effect 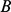 and thus, can have an impact on the power of the test for column effects as well as interactions, resulting in increased power for tests of both marginal effects and the overall model 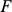. In the examination of the overall model 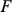 the 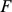 test is stochastically increasing when the interaction term is present, that is the value of the ESS for the whole model is greater under the epistatic model than the additive model. The total ESS for this scenario is 
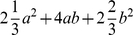
. Compared to the ESS in Figure 2 these ESS are larger and so the 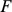 test of the full model will have greater power if this is the true scenario. Other cases of molecular epistasis are similar, and described in detail in the Methods Section. Epistasis can also change the power of the detection for a marginal effect of a different locus as can be readily seen by examining the ESS for the individual loci.

In Figure 1f, often considered the most diabolical case, the marginal effects are equal 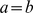. In this case, an increase in the row effect has no effect on the columns, but can increase the interaction. In the case of equal allele frequencies the wisdom of choosing the test of the full model is clear, as the only chance to detect this effect lies with testing the full model (main effects and interaction together) rather than the main effects alone. Upon close examination, it becomes clear that this is only true in the case where allele frequencies are equal, and when they are unequal, as in association mapping contexts, the tests for the marginal effects of one locus are affected by the distribution of the genotypes in the population which can in turn impact the 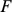 test for the individual loci. The ANOVA table for this case is given in the Methods Section.

### Simulations: estimated locus detection power as affected by epistasis

**Figure 4:**
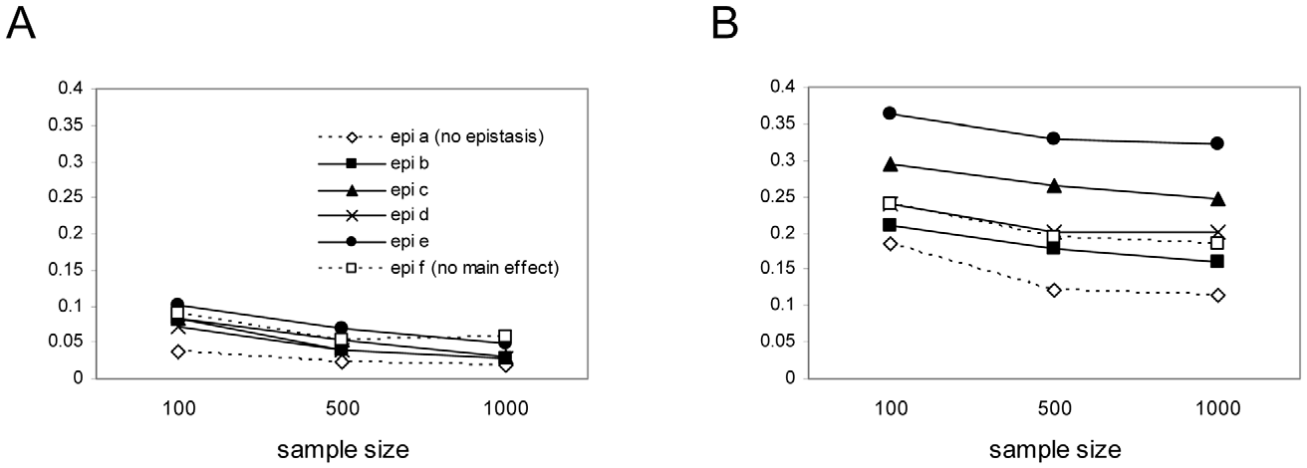
Goodness of fit for molecular epistasis. Proportion of variance explained by the QTL, estimated as the full model r2 of the correct model (averaged over 50 iterations). Panel A shows results for QTL with disparate allele frequencies (low minimum allele frequency); panel B shows results for QTL with more similar allele frequencies (high minimum allele frequency).

Many power studies for epistatic QTL are based upon the statistical models and statistical definitions of epistasis. In those cases data are simulated based upon the amount of variance explained by the genetic model, which is then further partitioned into main effect and interaction terms. However, molecular models of epistasis for a quantitative trait (Figure 1b–f) are more intuitively described in terms of relationships among means. This casting of the model in terms of relationships among means also indicates that in an ANOVA framework a fixed effects model can be employed as in Equation(1). The simulations conducted in this paper were all based upon distribution of means, and the impact of varying the relationships among means. One of the benefits of using the fixed effects model, and the corresponding cell means parameterization is that the total effect size (the distance between the means relative to the standard deviation) can be held constant, as is conventional in many statistical power analyses. The question then becomes: for a fixed effect size, how does epistasis impact the percentage of explained variance? Interestingly, epistasis tended to increase the proportion of variance explained by the correct model (Figure 4).

**Figure 5:**
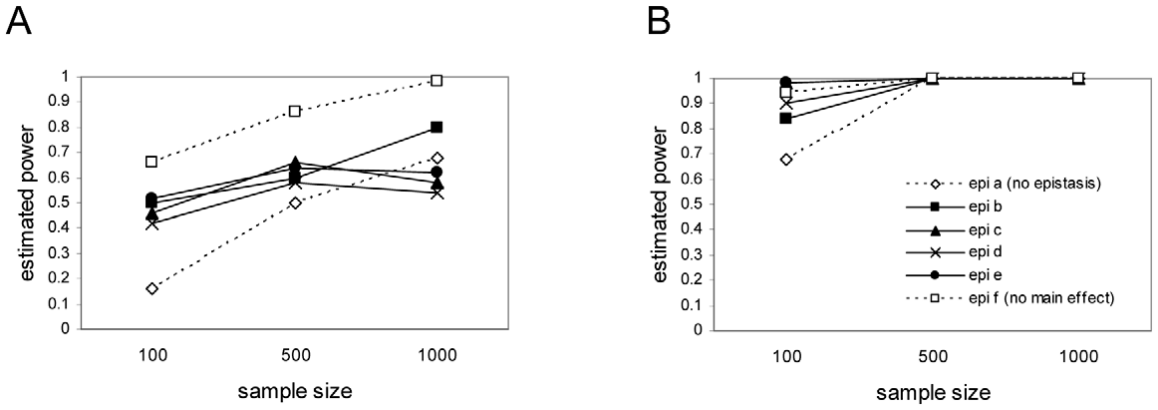
Power for detection. Power of detection for the different 2-locus models. Power was estimated as the proportion of iterations in which the full model F test for the correct model had a 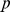 value 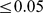. Panel A shows results for simulations with disparate allele frequencies within each locus (1%–10% for the rare allele and 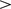
90% for the other allele); panel B shows results for simulations with allele frequencies within each locus closer to equal (20%–30% for one allele and 70%–80% for the other allele). Sample sizes were 100, 500, or 1000 diploid individuals.

This result implies that when the proportion of total variance is held constant under simulation conditions, as has been done in some studies, epistatic models then must often force the effect size simulated to decrease. Such approaches might fail to appreciate the true impact of molecular forms of epistasis on QTL detection power. In fact, detection power, estimated as the proportion of iterations in which the full model F test for the correct model had a *p* value below 0.05, was often higher in the presence of epistasis in our simulations (Figure 5). Over much of the parameter space highest power was achieved with extreme forms of epistasis. The model types with the highest 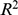 were not always the ones with highest power, for example epistasis form ‘f’ (Figure 1) does not have the highest 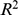, although it can have the highest power (Figure 5a).

The simulations demonstrate that epistatic models can be more powerful than the additive model (Figure 5). This is particularly apparent when one of the QTL alleles is rare. These results are consistent with recent reports [34], [39], [46], [47]. Under many forms of epistasis, the expectation is for higher ESS, in turn leading to higher noncentrality parameters of the 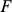-test and higher power than under additive models. As it is believed that many traits of biological interest are subject to epistasis, this is potentially good news for empiricists seeking to map trait-affecting loci.

### Simulations: detection of epistatic QTL in a full genome scan

**Figure 6:**
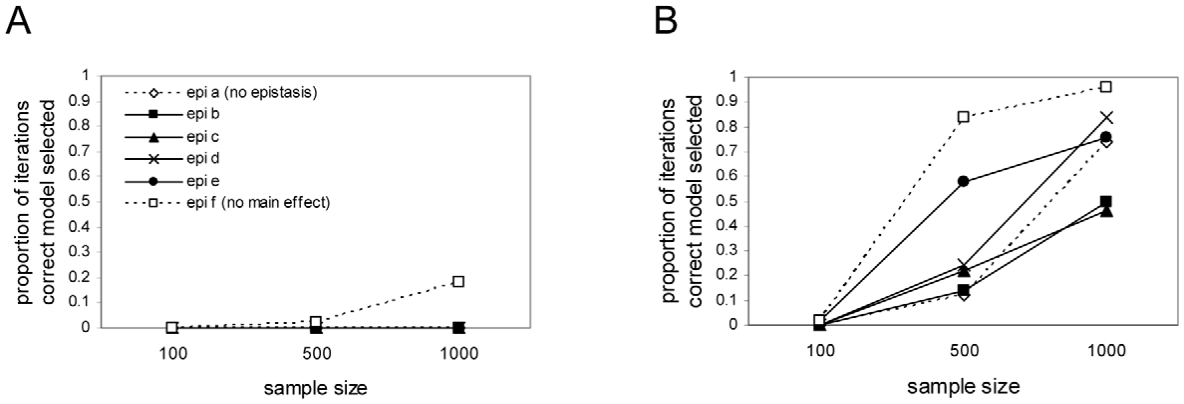
Selecting the correct model. Performance of the BIC model selection procedure: accuracy, estimated as the proportion of iterations where the selected model based upon BIC is the correct model without over- or underfitting. Within each panel the six types of epistasis are as in Figure 1. Panel A shows results for simulations with disparate allele frequencies within each locus (1%–10% for rare allele and 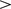
90% for the other allele). Panel B shows results for simulations with allele frequencies within each locus closer to equal (20%–30% for one allele and 70%–80% for the other allele).

Determining that the power of the correct model can be higher under epistatic conditions is only the first step. Realistically, single models are not fit in association mapping studies. Instead, genome scans of single and pairs of loci are conducted. The question then becomes: “How does the chance of selecting the correct loci depend upon the genetic model?”. A relatively straightforward grid search algorithm was used to search the model space and BIC was used as a model selection criteria. Reliable detection of the correct loci required large sample sizes and allele frequencies to be closer together than is often the case in association studies (Figure 6). Particularly disheartening was the low probability of selecting the correct loci at sample sizes of n = 1000, when allele frequencies are disparate.

**Figure 7:**
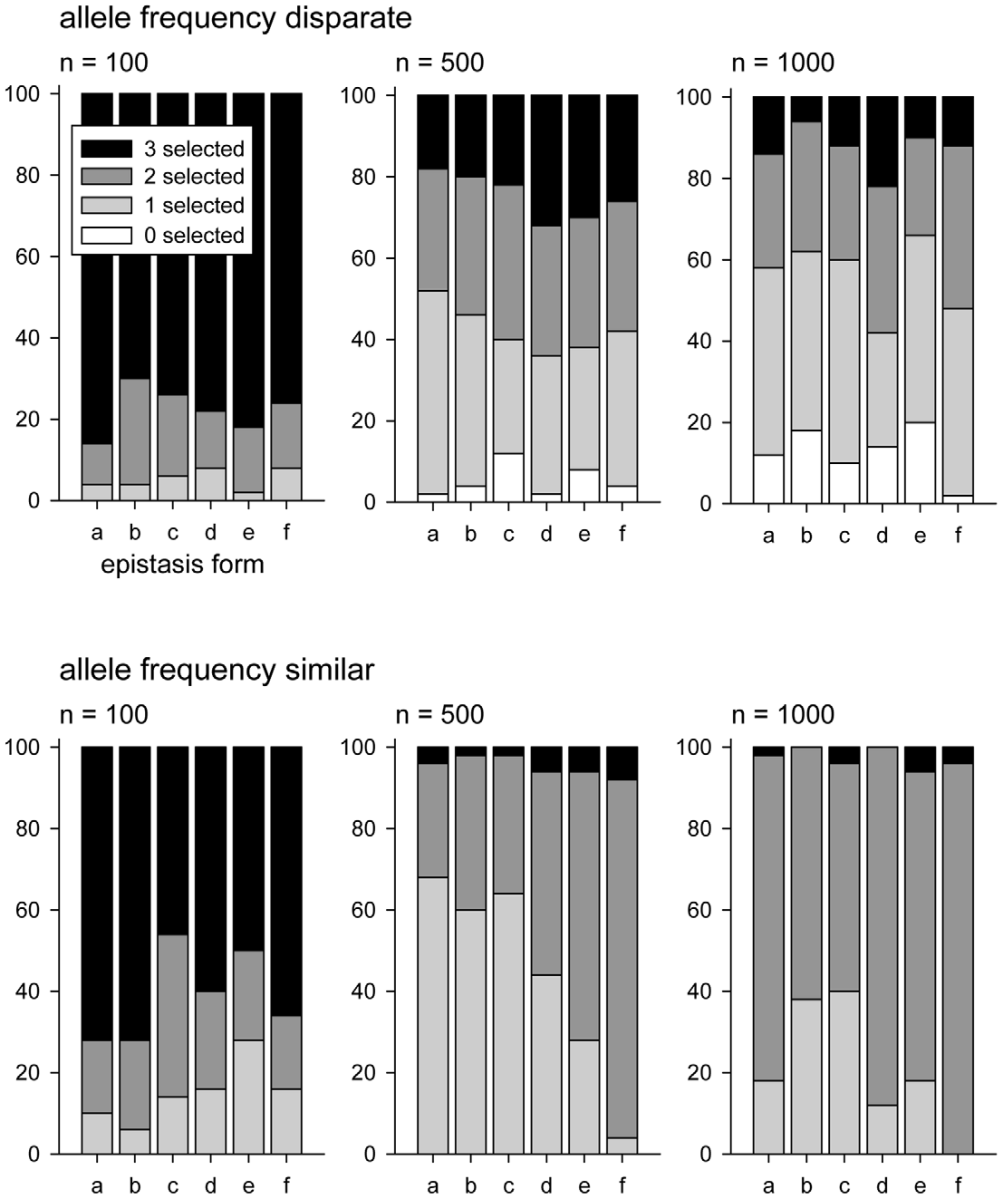
Performance of the BIC model selection procedure: number of loci selected. For each simulation setting, the percentage of iterations is given in which 0, 1, 2, or 3 loci were selected. Within each panel the six types of epistasis are as in Figure 1. Upper panels show results for simulations with disparate allele frequencies within each locus (1%–10% for rare allele and 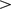
90% for the other allele); lower panels show results for simulations with allele frequencies within each locus closer to equal (20%–30% for one allele and 70%–80% for the other allele).

**Figure 8:**
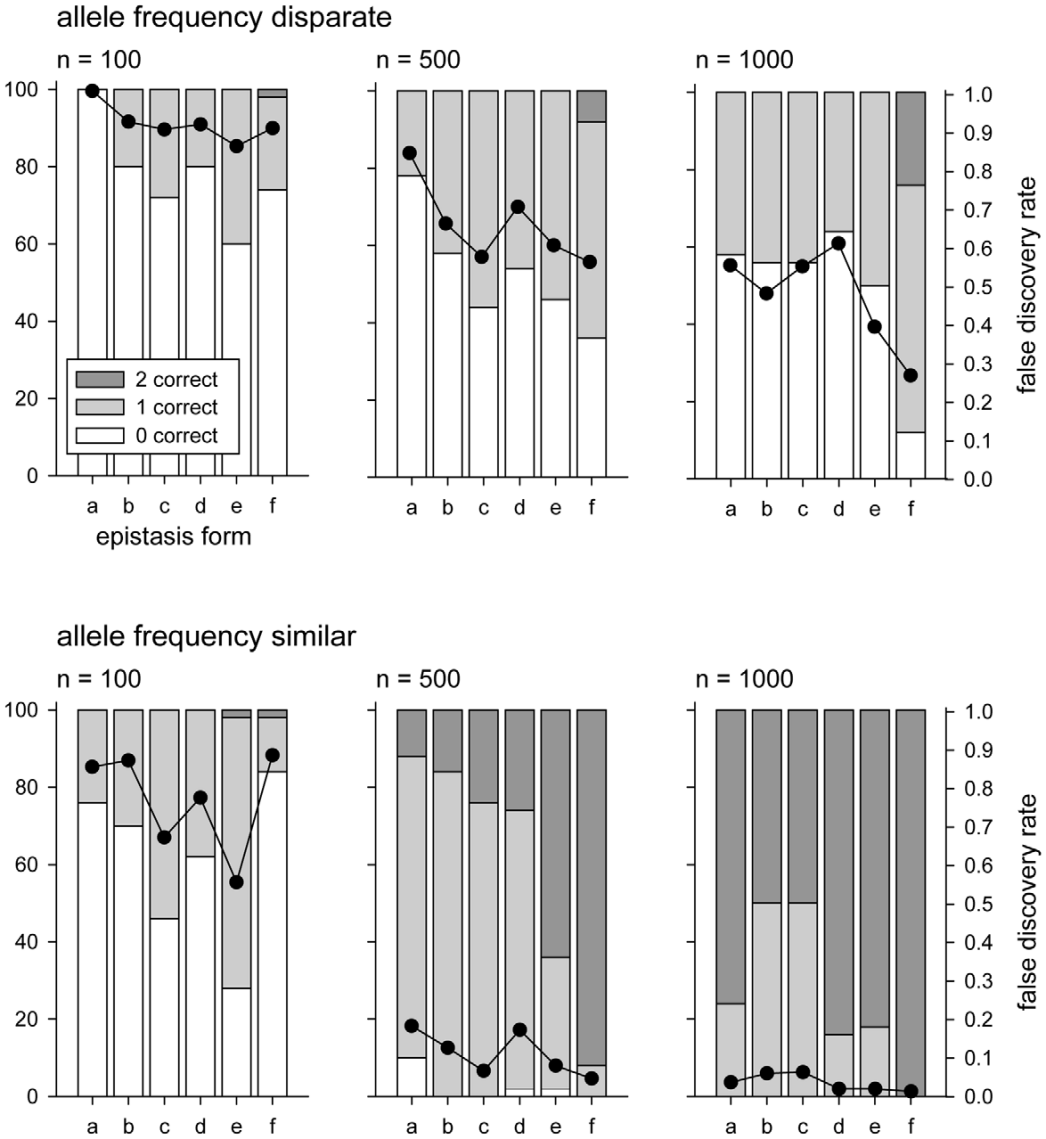
The number of selected loci that are correct. Performance of the BIC model selection procedure: number of specified loci that are selected correctly. For each simulation setting, the percentage of iterations is given in which 0, 1, or 2 of the specified QTL loci were correctly identified (stacked bars, left Y-axis). Also shown is the observed false discovery rate, defined as the average proportion of selected loci that were not specified QTL (dots, right Y-axis). Within each panel the six types of epistasis are as in Figure 1. Upper panels show results for simulations with disparate allele frequencies within each locus (1%–10% for rare allele and 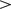
90% for the other allele); lower panels show results for simulations with allele frequencies within each locus closer to equal (20%–30% for one allele and 70%–80% for the other allele).

Small sample sizes tended to result in overfit models, rather than underfit, selecting more than 2 loci (Figure 7), as is expected with BIC [55]. Moreover, even when the correct number of loci were selected, the actual loci selected were often not the causal locus (Figure 8). When allele frequencies were disparate, it was extremely rare to correctly identify both loci contributing to the trait, even when sample sizes were large. The outlook was less bleak when allele frequencies were closer together and sample sizes were large: at least one, and often both, of the specified QTL were correctly identified (Figure 8).

Interestingly, in many cases the presence of epistasis tended to increase the performance of the model selection. For example, when allele frequencies are similar and sample size is low (n = 100), several forms of epistasis lead to a higher probability of correctly detecting at least one of the specified QTL, and also to lower false discovery rates, compared to the additive case. The same pattern is true at moderate sample sizes (n = 500). When sample sizes are large (n = 1000) the additive model outperforms epistatic models 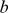 and 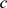, but epistatic models 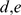 and 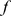 are still more likely to correctly select the specified QTL. One possible explanation, is that in the additive case the correct model is not included in the search space when only full models are examined.

When allele frequencies are disparate, as is likely to be the case for most association mapping studies, the pattern is more subtle. In all sample sizes considered the epistatic models appear to have a slight advantage in selecting the correct loci but given the size of the simulation study, this can not be said to be a statistically significant difference. Fortunately, when sample sizes are reasonable (n = 500, 1000) and allele frequencies are closer together incorrect loci are unlikely to be selected (Figure 8, lower panels).

These model selection results illustrate two points. First, detection of loci using BIC-based model selection results in a high proportion of model misspecification in low-power situations (small sample size, low allele frequencies) but yields good results (low proportion of false discoveries and one or both of the specified loci correctly identified) with reasonably large sample sizes and similar allele frequencies. Second, and most important, the presence of epistasis does not appear to reduce detection efficiency but, in contrast, increases detection rates over much of the parameter space. This is consistent with the power estimates (Figure 5) and the predictions based on the balanced model.

When allele frequencies are closer together, as in many cross-based experimental QTL studies, the power for detection of loci is good among all models once the sample size is larger than 500 total (Figure 5). Power even in sample sizes of 1000 is substantially lower when allele frequencies are disparate. Selecting the correct model is also more challenging when allele frequencies are disparate (Figure 6), as is estimating the number of loci contributing to the trait (Figure 7). Only when allele frequencies are similar, and sample sizes are relatively large (n = 1000) is model selection straightforward.

Our simulations show that the presence of epistasis between two QTLs can increase QTL detection power compared to the additive case where the two QTLs control trait variation independently of each other. The power of single-locus scans versus multilocus full model scans, when epistasis is present, has been addressed in previous studies (e.g. [39], [47]); these studies showed that two-locus full model scans outperform single-locus scans. We show that the presence of epistasis potentially increases statistical power to detect underlying QTLs, and does not decrease the power of detection, leading us to the conclusion that epistasis is not necessarily an obstacle that complicates QTL detection efforts.

One issue that is unexplored is what model selection strategy is optimal for cases with and without epistasis, and whether the optimal strategy differs when epistasis is present or absent. Is there a possible negative impact of multiple locus scans, when epistasis is not present? In theory, at infinite sample size, model selection using BIC selects the true model if it is included in the search space [56]. When loci are independent, the tests within the multiple locus model are identical to the tests in a single locus model [57]. When no interaction is present the inclusion of the interaction term will not affect the model fit, although there will be a difference in the degrees of freedom. In small samples, however, it is unclear how model selection is affected if BIC is the criteria used for model selection and the true model is not part of the model search space. In the context of our simulations the question is if, or to what extent, failing to include the model without the interaction term in the search space results in reduced likelihood that the best model includes the two QTLs. An additional set of simulations were performed to gain insight in this issue (data not shown) using datasets generated by an additive genetic model (two QTLs without interaction; Figure 1a) and comparing model selection approaches that search all 1-, 2-, and 3-marker models that include interactions between markers (full models) and without interactions (main effects models). In all explored cases both search approaches performed equally well in identifying the two QTLs in the best model. However, in a minority of cases overfitting (i.e., selecting a best model with 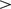
2 markers) was more frequent in the ‘full model’ search. Importantly, the correct markers were identified in the overfit models. It must also be recognized that other criteria such as AIC (Akaike Information criteria) and informed searches [46] rather than exhaustive searches, may also perform better than the simple approach discussed here. The main finding is not that our particular model selection strategy is optimal in all cases, but rather that in the implementation of a common model selection strategy, epistasis does not necessarily harm your chances of picking the best model.

Detection of loci underlying quantitative traits efforts can benefit from accounting for marker interactions when epistasis is in fact present [39], [46], [47]. Empirical results show that the presence of epistasis can increase power to detect the underlying loci compared to the non-epistatic additive case, in two-marker full model tests as well as in single-marker main effect tests. This was confirmed via simulation, where locus detection via fitting full models was often facilitated by the presence of epistasis. As it is becoming increasingly clear that epistasis is a crucial component in many biological systems, these results indicate that epistasis may not have the negative impact perceived by many biologists, and that detection efforts may in fact benefit from the presence of epistasis.

## Methods

### Statistical model

For the twoway fixed effects model with interaction, the usual model equation is
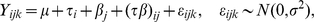
(1)

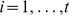
, 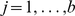, 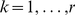.

It will be simpler to rewrite this model in the equivalent *cell means model*

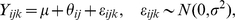
(2)
**where we can equate 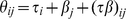. From this model we can construct an ANOVA table with expected sums of squares (Table 2), which parameterizes the two way ANOVA model in terms of the classic factor effects model and contains the parameters representing the marginal effects of Loci 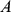 and 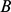 and the interaction among these loci. The third column of Table 2 gives Expected Sums of Squares (ESS) rather than the more common Expected Mean Squares. Statistically they are equivalent, with the ESS typically being a bit more transparent in calculation.

**Table 2:** Factors and Expected Sums of Squares for the twoway fixed effects cell means model.

Source	df	ESS
A	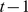	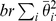
B	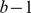	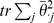
Epistasis	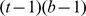	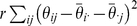

*The term involving the model error* 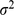 *, is omitted as it is present in all models.*

The ESS shown in Table 2 can be used to derive the power of the 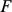 tests associated with these models. The ESS is proportional to the noncentrality parameter of the noncentral 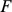, and the 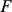 statistic has a monotone likelihood ratio in this parameter (see [58] Section 7.13). Simply put this implies that the power of the 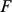-test will then increase as the noncentrality parameter increases. To compare two scenarios consider the following: for two configurations of parameters, denoted by 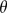 and 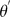, if
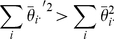
then the power of the 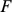 test for the main effect 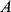 in the “
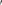
” configuration is greater.

This equivalent model is simpler in the sense that the identifiability restrictions are more obvious - only the assumption that 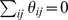 is needed to be able to estimate all effects. In particular, the main effects and interaction parameters are now given by
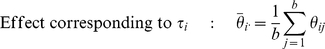


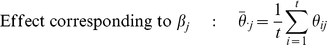


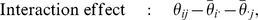
and recall that the identifiability constraint is 
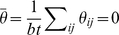
, and Table 2 shows that the epistasis effect contains the parameters of the marginal effects of 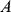 and 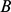. The question of interest is how these effects are intertwined.

First, note that if there is no interaction then one locus cannot influence another. To see this, recall that we have the identity 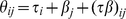. If there is no interaction then 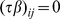 for all 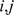, and 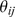 is of the form 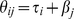, and the ESS are as in Table 3.

**Table 3:** Factors and Expected Sums of Squares for the twoway fixed effects cell means model when there is no interaction.

Source	df	ESS
A	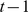	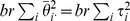
B	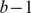	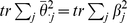
Epistasis	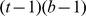	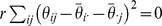

*Note that the identifiability constraint* 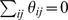 *implies* 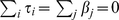 *. The term involving the model error* 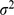 *, is omitted as it is present in all models.*

**Figure 9:**
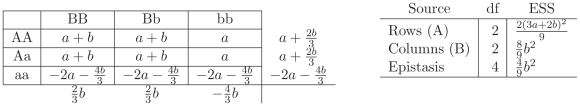
Punnett Square, Factors and Expected Sums of Squares for epistasis case c (**Fig. 1c**).

**Figure 10:**
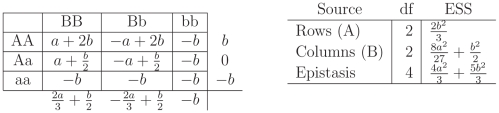
Punnett Square, Factors and Expected Sums of Squares for epistasis case d (**Fig. 1d**).

**Figure 11:**
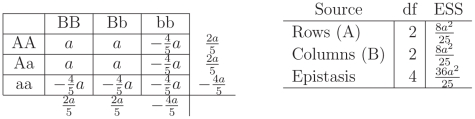
Punnett Square, Factors and Expected Sums of Squares for epistasis case e (**Fig. 1e**).

**Figure 12:**
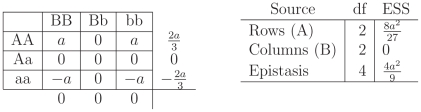
Punnett Square, Factors and Expected Sums of Squares for epistasis case f (**Fig. 1f**).

For the patterns in Figure 1:

- This is a no interaction pattern, which is addressed in Figure 2. With no interaction the marginal effects are independent, and changes in one margin have no effect on the other margin.
- This is addressed in Figure 3, and here the effect of epistasis clearly impacts the marginal distributions as well as the overall ESS. The overall ESS for this configuration is greater than the ESS for configuration a indicating that the non-centrality parameter for the 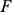 test for this scenario is stochastically greater than for the additive model.
- In this pattern recessive epistasis is combined with dominance. An increase in the column effect 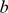 can produce an increased ESS and power in the row effect 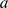 effect (Figure 9). It is also interesting to note that in this pattern the interaction (epistasis)is only dependent on the 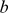 effect.
- In this pattern both loci exhibit recessive epistasis. The 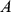 loci will only appear significant through an increase in the column effect 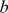, while the row effect 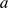 only influences the significant of the 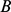 loci. Both row and column effects influence epistasis (Figure 10).
- This is recessive epistasis of the aa and bb combinations combined with dominance at the A locus. This is a highly restrictive pattern as there are only two distinct parameter values. With the constraint that the values sum to zero, there is only one value 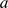 that is needed to specify all the means for this mode (Figure 11). Changes in one locus affect the other locus and the epistatic term.
- This case has been previously discussed in the results, the detailed ANOVA shows that 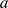 has no effect on the columns and, in fact, the column (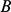 locus) has no main effect. The 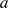 effect can increase the interaction (Figure 12). In a sense there is really no epistasis here (as there is only one real effect), but an analysis could find significant epistasis.

### Simulated datasets

In order to mimic populations of individuals likely to be encountered in testing for association in natural populations, the coalescent model was used to generate random, diploid samples drawn from a large, random mating population of constant size. Assuming neutral evolution, the coalescent process models the history of a population sample of sequences [59] by randomly generating a possible genealogy for the sequences. The process starts from the present-day sample and proceeds backward in time, allowing a succession of coalescent and recombination events to shape the genealogy. The waiting times between successive events and their relative probabilities are functions of the number of lineages present and the recombination rates between loci. A coalescent event joins two randomly chosen lineages into one common ancestor. A recombination event splits one randomly chosen lineage in two, with different ancestors for the two sequence segments on either side of the recombination position. The process continues until all lineages have coalesced in one individual, the most recent common ancestor of the entire sample. Subsequently, marker mutations are superimposed on the branches of the genealogy according to a specified mutation model (*e.g.* biallelic SNPs). The genealogy with its defined mutation positions defines the SNP haplotype sequences of the sample.

Samples were simulated based on a coalescent model with recombination [60], [61]. This process was modified to permit the simulation of thousands of loci at fixed recombination distances by exploiting the sparsity of the Markov Chain structure. In this way recombination rates between each pair of adjacent loci can be specified, and multiple recombination events are permitted at any given position [62]. The program can be downloaded from http://www.stat.purdue.edu/~simonsen/simcore/.

Simulated samples consisted of 100, 500 or 1000 diploid individuals, with individual genomes consisting of 5 independent linkage groups with 100 biallelic SNP markers per group. An equal population recombination rate (2Nr) of 1 between adjacent markers was specified. This can be thought of as marker distances of 5 kb for a species with an effective population size of 10,000 and a uniform individual recombination rate of 1% per Mb (which is considered a simplified model for humans [63]). Mutations at the 500 specified marker positions were superimposed randomly on branches of the generated genealogies, with a branch's probability of attracting mutations proportional to its length. When appropriate (see below) the minimum allele frequency for designated SNPs was manipulated to a desired range by allowing the mutation to occur only on those branches that would lead to a suitable number of descendants.

Two markers (on positions 50 and 150) unlinked to each other were designated as loci affecting a hypothetical quantitative trait. For each individual a trait value was generated based on a specified genetic model and the individual's genotype, adding an error drawn from a z distribution (SD = 1). We considered two-locus genetic models that either showed no epistasis (Figure 1a) or one of several forms of molecular epistasis (Figure 1b–f). Locus effects were modeled via cell means parameterization, in which genotypic trait means for all nine possible genotypes were specified. The cell means parameterization is equivalent to the more common factor effects parameterization [57] but is more convenient for simulating specific epistatic scenarios. To compare the different 2-locus models we maintained a fixed range of genotypic means for all models. The difference between the smallest and the largest of the nine trait means was fixed at 1.6 SD. Note that in typical power analyses [64] the power is derived as a function of the effect size (the difference between the means/standard deviation), the type I error and the sample size. We keep the effect size between the smallest and largest mean constant to determine the effect of epistasis on the power for this fixed phenotypic range. In order to compare results to some QTL type studies, we also calculate the proportion of variance explained by the correct model in each simulation.

For each parameter setting 50 replicated simulations were performed. In order to avoid unwarranted stochastic genealogical variation between simulations we held the underlying genealogies (but not mutations) constant while varying locus frequency, epistasis type and locus effect size.

In the simulations we manipulated allele frequencies at the two loci, while frequencies at the 498 other marker loci were unconstrained. Differences in allele frequency are important to consider because (1) they affect sample sizes (and thus detection power) for the nine possible genotypic classes in the population; and (2) with epistasis, the translation of biological epistasis into statistical epistasis is allele frequency dependent. That is, a given epistatic case from Figure 1 can give rise to different partitioning of the total genetic variance into main effects and interaction effects, and this depends on locus allele frequencies [*e.g.* 39]. In our biallelic loci, by convention we specify the smaller of the two allele frequencies and call this the minimum allele frequency. Minimum allele frequencies at the loci were constrained to either ‘low’ (1–10%) or ‘high’ (20–30%). In the case where the minimum allele frequency is low, the difference between the frequencies of the two alleles at one locus is large and disparate, while the differences are much smaller when the minimum allele frequency is high. Note that the effect of locus frequencies in association mapping studies differs from typical QTL linkage mapping in experimental populations, where marker frequencies are under experimental control due to breeding designs and are often equal among alleles.

We evaluated how QTL detection power is affected by epistasis by fitting the true model in each simulated dataset and tabulating how often the full model *F*-test was significant. Type I error rates were examined by fitting all 1-, 2-, and 3-marker models using only markers that were unlinked to any causative locus. Ten evenly spaced markers on each of the three chromosomes that did not contain a locus with an effect on the trait were selected and all 4525 possible models using these 30 markers were fitted. Averaged over iterations, the proportion of models with a full model F test that had a *p* value below 0.05 was at or below the nominal level for all simulations (data not shown).

### Model selection procedure

To identify markers associated with the phenotype in a genome-wide scan a 2-step model selection procedure was followed. The first step aimed to reduce the dimensionality of the dataset by selecting a subset of informative markers for further analysis. At this point no block was eliminated from consideration and the purpose of this step was purely to reduce the number of models tested in the next step to a set that is computationally tractable, without losing information in the model space. The approach is to temporarily discard markers that are correlated to other markers due to physical linkage (which, in the absence of recombination, causes markers to be inherited together and generates population-level associations between alleles at nearby markers [65]). The approach is similar in spirit to Coffman *et al.* [46] who reduced marker datasets in cross-based QTL analysis by pre-selecting only one (the ‘best’) marker per linkage group. To accomplish this, genomes are parsed into phylogenetically compatible blocks, for which genealogies could be inferred without invoking recombination. Within each block all one- and two-marker models were evaluated for association with the phenotype; the markers from the best model (based on the Bayesian Information Criterion (BIC) values) were selected for further consideration.

The second step evaluated the overall 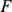-test for all one-, two-, and three-marker models that could be constructed with the reduced set of quasi-independent markers. Sorting models based on their BIC value, the markers from the best model as loci identified by the selection procedure were identified. In summarizing the results over 50 iterations, we looked at the proportion of simulations that correctly identified zero, one, or both of the QTL loci that were specified; the total number of loci that were selected; the proportion of iterations that correctly identified the specified model; and the average proportion of selected loci that were incorrect (false discovery rate). In evaluating the model selection results only those cases that identified the specified loci exactly were considered correct hits. Markers that are on the same chromosome as a causative locus can be in linkage disequilibrium with the causative locus due to shared inheritance of physically linked markers. Selection of linked markers by the model selection procedure may correctly identify the presence of the genetic effect even when the exact position is not identified. By considering only the exact specified locus as correct hits this represents a lower bound on the detection power. Note that many applications use a significance test for model selection, and in that case the proper distribution needs to be carefully considered.
